# Reproducibility of #Enzian classification by transvaginal ultrasound and its correlation with symptoms

**DOI:** 10.52054/FVVO.16.1.008

**Published:** 2024-03-28

**Authors:** C Russo, L Lazzeri, T Siciliano, A Selntigia, D Farsetti, C Chiaramonte, F.G. Martire, E Zupi, C Exacoustos

**Affiliations:** Department of Surgical Sciences, Obstetrics and Gynecological Unit, University of Rome “Tor Vergata”, Italy; Department of Molecular and Developmental Medicine, Obstetrics and Gynecological Clinic University of Siena, Italy; Department of Statistics, University of Rome Tor Vergata, Italy

**Keywords:** Endometriosis, #Enzian classification, transvaginal ultrasound, endometriosis staging, endometriosis symptoms

## Abstract

**Background:**

The #Enzian classification represents a system to describe endometriotic lesions during surgery. Its use is well established in correlating ultrasound and surgical findings.

**Objectives:**

To describe interobserver reproducibility of ultrasound use and symptom correlation with compartments involved using #Enzian classification.

**Materials and Methods:**

Two experienced operators performed transvaginal sonography (TVS) in 52 patients affected by pelvic endometriosis. A rate agreement was determined. A further 200 women with endometriotic TVS signs, with no previous surgery and not taking any hormonal therapy, were staged by one of three different operators according to the #Enzian (compartments A, B, C, O, T, FA, FB, FI, FU, FO). Statistical analysis compared all the compartments, as single or associated, with single or combined symptoms (dysmenorrhea, dyspareunia, heavy menstrual bleeding - HMB, bowel symptoms).

**Main outcome measures:**

Evaluation of the reproducibility of #Enzian classification in assessing pelvic endometriosis among different operators using TVS, and of possible associations between symptoms and specific #Enzian compartments.

**Results:**

Excellent agreement between the two operators in evaluating almost all the compartments (k >0.8) was observed. Dysmenorrhea did not correlate with any specific compartment. We observed a significant association between dyspareunia and B compartment (p=0.02). HMB is associated with FA (p=0.02). Bowel symptoms were associated with B (p=0.02). Combining more symptoms, we observed more significant associations with different compartments.

**Conclusions:**

#ENZIAN classification is reproducible in the evaluation of pelvic endometriosis. Some symptoms are correlated to specific ultrasound signs of the disease.

**What is new?:**

An accurate evaluation of symptoms could guide TVS examination to detect specific endometriotic lesions and establish the best management for the patients.

## Introduction

Endometriosis affects about 10 to 15% of the female population (Viganò et al., 2007). Endometriosis symptoms can range from asymptomatic to incapacitating pain. It is associated with infertility in 30-50% of cases (Rogers et al., 2013). In women suffering from endometriosis, diagnosis and treatment have enormous relevance to quality of life. Therefore, it is crucial for clinicians dealing with this condition to systematically classify and reproduce the findings obtained in each case.

The traditional classification systems of endometriosis, developed by several professional organizations worldwide (AFS, rASRM, Enzian, rEnzian, AAGL), have been historically based on surgical findings. (Revised American Fertility Society classification of Endometriosis, 1985; Revised American Society for Reproductive Medicine classification for endometriosis, 1997; Haas et al., 2013a; Abrao et al., 2021).

In 2019, a group of experts released the new #Enzian classification system, which includes all the anatomical locations of pelvic endometriosis, grading lesions according to size, describing adhesions, and other organs involvement. This system was thought of as a surgical classification and subsequently applied to presurgical diagnostic imaging. (Keckstein et al., 2021).

Several published papers demonstrated the high accuracy of ultrasound imaging in identifying features of endometriosis (Leonardi et al., 2020; Hudelist et al., 2021; Enzelsberger et al., 2022; Indrielle-Kelly et al., 2022); moreover, recent papers reported how the use of the #Enzian classification in an ultrasound setting correlated well with the classification after surgery (Di Giovanni et al., 2022; Montanari et al., 2022; Bindra et al., 2023).

The high agreement between ultrasound and surgery is also mentioned in the recent ESHRE guidelines (Becker et al., 2022), which highlight that imaging can be utilised to diagnose endometriosis without the need for diagnostic surgery.

The association between symptoms and classification has been extensively investigated, given that the management of endometriotic patients is based on both the localisation and size of the lesions, as well as the symptoms reported by the patients (Fauconnier et al., 2002; Haas et al., 2013b; Perellò et al., 2017; Montanari et al., 2019; Morgan- Ortiz et al., 2019).

However, these studies still reported surgery as the gold standard for diagnosis of endometriosis.

In this study we decided to perform, after an accurate evaluation of the symptoms, a careful non-invasive staging of pelvic endometriosis by TVS according to #Enzian classification; this could help the physician to adequately detect the lesions in different pelvic compartments in order to plan medical or surgical treatment.

Since most of the patients still have a surgical diagnosis, often with associated incomplete surgery, or take hormonal treatment, which could alter the real symptomatology of the disease, we decided to investigate by TVS only patients with ultrasound signs of endometriosis who have not undergone previous surgical or hormonal therapy.

Based on the aforementioned points, the aim of this study was to describe ultrasound feasibility and interobserver reproducibility of the #Enzian classification in patients who had never undergone surgery and/or hormonal treatment, and to correlate endometriosis symptoms to #Enzian compartments, by using a non-invasive ultrasound diagnosis. *Ethical approval:* Institutional review board approval was obtained (No. 6/19). All patients signed the informed consent.

## Materials and Methods

### Settings and participants

The study was carried out at the Gynecological Unit (Department of Surgery Obstetrics and Gynecological Clinic) of the University of Rome “Tor Vergata”, from January 2020 to September 2022.

A total of 200 women with suspected pelvic endometriosis were prospectively enrolled in the study. Inclusion criteria were the presence of at least one ultrasound feature of endometriosis, premenopausal status, no hormonal therapy for at least 6 months, no previous abdominal surgery, no ongoing pregnancy, no diagnosed gastrointestinal diseases and no malignancy of the reproductive tract.

Exclusion criteria included being post-menopausal, previous abdominal surgery, current use of hormone therapy or who have taken hormonal treatments within six months of enrolment, pregnancy, gastrointestinal diseases and known reproductive tract cancer or other pelvic pathologies that could interfere with an accurate ultrasound examination (such as large fibroids or pelvic masses).

The pelvic endometriosis of these patients was classified according to the #Enzian classification. For all patients, symptoms were recorded and evaluated by a visual analogue (VAS) score. Symptoms type (with a VAS score ≥5) and #Enzian compartments were correlated in the statistical analysis.

To evaluate the feasibility and reproducibility of the #Enzian classification, 52 more patients underwent TVS real-time examination by two experienced sonographers (C.E. observer A, and L.L. observer B). Observers A and B are both dedicated to gynaecological ultrasound (both with more than 10-year experience). All women underwent the ultrasound examination by the first observer A and immediately after by the second observer B. The second observer was unaware of the findings and measurements of the first observer. The two operators were not blind to the patient`s history, physical examination and symptoms thus enabling better evaluation during the transvaginal examination for the ultrasound signs of pelvic endometriosis.

### Clinical history and symptoms

Patient information was obtained by carrying out a personal anamnesis, based on a pre-established format on File Maker pro® Version 9.0 software. The collected data included: age, BMI, gravidity, and parity. The presence of dysmenorrhea, dyspareunia, dysuria and bowel symptoms were evaluated with a VAS system by personally conducted questions by the clinician; VAS score consists of a 10 cm line, ranging from 0 as no pain and 10 as the worst experience of pain. Symptoms were considered present when the VAS score was ≥5 (Exacoustos et al., 2022).

Patients who reported at least one symptom from dyschezia, diarrhoea, constipation, alternating symptoms between constipation and diarrhoea, rectal tenesmus and rectal pain/colic were classified as having bowel symptoms. (Fauconnier et al., 2002; Martire et al., 2023) The amount of menstrual bleeding was assessed through subjective evaluation of the patient and the presence of heavy menstrual bleeding (HMB) was specified. This subjective evaluation is reported in the literature as reliable and comparable with the pictorial blood loss analysis chart score (PBAC) (Higham et al., 1990).

### Ultrasound examination

Transvaginal ultrasound examination was performed by three different operators (C.E, C.R., F.G.M.), with either a GE E8 or E6 (GE Healthcare) ultrasound machine, using a wideband 5–9-MHz endocavitary 3D transducer. The TVS examination was performed at any phase of the menstrual cycle. The ultrasound settings, both 2D/3D grayscale and Doppler were standardised and identical for all subjects.

All possible locations of endometriosis were evaluated and recorded using previously published mapping sheets (Guerriero et al., 2016; Exacoustos et al., 2014). The pelvis was investigated in the anterior, lateral, and posterior compartments, and deep infiltrating endometriosis (DIE) lesions of the bladder, ureter, parametria, posterior vaginal fornix, uterine torus, uterosacral ligaments, rectovaginal septum (RVS), and the lower and upper rectum were considered for this study (Guerriero et al., 2016; Exacoustos et al., 2014).

#### #ENZIAN Classification System

According to the #Enzian classification, the small pelvis is divided into three compartments for DIE: compartment A comprises the RVS and vagina, including the torus uteri; compartment B comprises the uterosacral (USL) and cardinal ligaments, parametria, and pelvic sidewalls; compartment C comprises the rectum (defined as up to 16 cm from the anal verge) In addition, further locations (F) are classified as adenomyosis (FA), urinary bladder involvement (FB), or ureteric involvement with signs of obstruction (FU). Bowel disease cranial to the rectosigmoid junction (FI, >16 cm from the anal verge; sigmoid colon, transverse colon, caecum, appendix and small bowel) and other extragenital locations such as the abdominal wall, diaphragm and nerves were also included. All these other extragenital locations were taken together and described as #Enzian compartment FO. Finally, peritoneal disease, ovarian endometriosis and pelvic adhesions (including the Fallopian tubes pathological conditions) are listed as P, O, and T. According to the #Enzian classification, tubal patency can optionally be evaluated using TVS and may be recorded as part of #Enzian compartment T. Data on tubal patency were not included in the present study. Peritoneal endometriosis (P) is not easy to detect by TVS and it can be suspected only by indirect ultrasound signs; therefore, it was not considered in the ultrasound evaluation.

#### Ultrasound evaluation of different compartments

**Compartment A** is a vertical plane extending from the pouch of Douglas and encompasses the vagina and RVS. Severity is graded by measuring the largest diameter of the lesion: Grade A1 ≤1 cm, Grade A2 1–3 cm, and Grade A3 >3 cm. The measurements of the lesions are taken from the posterior vaginal fornix, the rectovaginal septum, and the torus uteri without measuring the tissue infiltrating the rectal wall.

**Compartment B** is a horizontal lateral plane including the USL, the parametria, and the pelvic sidewall. The measurements of the lesion are taken from the posterior and lateral retrocervical areas without measuring the tissue that infiltrated the rectal wall. The right and left sides will be recorded separately. Severity is graded by measuring the largest diameter of the lesion: Grade B1 = <1 cm, Grade B2 = 1–3 cm, Grade B3 = >3 cm.

**Compartment C** is located dorsally and expresses the spread of the disease to the lower rectum and rectum/sigmoid walls. The measurements should be the largest diameter along the rectal wall (longitudinal diameter). In the case of multifocal lesions, the length of all the affected bowel walls is considered, in the case of multicentric lesions (distance from two lesions more than 2 cm or different bowel segments) the largest lesion should be considered. Extension of the lesion is graded measuring according to the largest diameter: Grade C1 ≤1 cm, Grade C2 1–3 cm, and Grade C3 >3 cm.

**Compartment T** considered the number of pelvic adhesions between pelvic organs. They are described as follow: T1 = adhesions between the ovary and pelvic sidewall +/− tubo-ovarian adhesions; T2 = T1 plus adhesions to the uterus or isolated adhesions between the adnexa and uterus; T3 = T2 plus adhesions to the USL and/or bowel or isolated adhesions between the adnexa and the USL and/or bowel. Adhesions were suspected during the TVS examination if the ovaries and/or the uterus appeared fixed to the adjacent structures while the sonographer conducted abdominal palpation.

**Compartment O** considered endometriosis of the ovaries. Endometriomas are described in size and location: O1 = <3 cm, O2 = 3–7 cm, O3 = >7 cm. In case of multiple endometriomas the sum of the maximal diameter of all endometriomas should be considered. Measurements in three orthogonal planes (longitudinal, anteroposterior, and transverse) for each endometrioma were recorded.

**Compartment FA** considered uterine adenomyosis: all possible sonographic findings of uterine adenomyosis (Exacoustos et al., 2011; Van den Bosch et al., 2015) were evaluated. The 2D examination was followed by the acquisition of 3D data using the 3D volume mode. The type of adenomyosis was defined as either diffuse, focal and adenomyoma (Lazzeri et al., 2018). The extension of each type of adenomyotic lesion in the external myometrium and the junctional zone was divided into four grades according to a previously published scoring system (Lazzeri et al., 2018).

**Compartment FB** considered vesical endometriosis. Bladder DIE is diagnosed only if the muscularis of the bladder wall is affected.

**Compartment FU**. In cases of ureteral DIE, FU correlates to obstruction-related dilatation in a funnel shape either caused by a lesion corresponding to DIE (usually parametrial origin) (Guerriero et al., 2023) or by DIE of the urinary bladder with or without hydronephrosis; it is not graded because it is, by definition, a severe status. In cases of diagnosis by TVS, a ureteral diameter ≥6 mm is interpreted as a congestion sign correlating to ureteric obstruction (Carfagna et al., 2018; Keckstein et al., 2021).

In all women with DIE, a transabdominal scan of the kidney to search for ureteral stenosis is necessary, because the prevalence of endometriotic lesions in the urinary tract may be underestimated and women with DIE involving the ureter may be asymptomatic. (Pateman et al., 2015). The degree of hydronephrosis should be assessed and graded using the generally accepted ultrasound criteria (Knabben et al., 2015).

**Compartment FI** defined intestinal disease cranial to the rectosigmoid junction. These lesions are difficult to diagnose by ultrasound. Ileo-cecal DIE can be seen or at least suspected when adhesion or endometriosis is seen on the right adnexa.

**Compartment FO** considered other locations, such as the abdominal wall or diaphragmatic endometriosis. Transabdominal ultrasound can see quite accurate lesions of the abdominal wall. For other types of lesion MRI may be requested.

### Statistical analysis

All statistical analyses were carried out using Medcalc version 9.2.0.2 (Medcalc Software, Mariakerke, Belgium). Measurements concerning quantitative variables were developed with arithmetic mean and standard deviation (SD). The qualitative or categorical variables were expressed as the number of cases (n) and relative rate (%). The inter-observer agreement for classifying the presence or absence of endometriosis in the different compartments and the 3 grades was evaluated with the Cohen K index using a the 95% confidence interval. General rules for the interpretation of k coefficients were used, i.e. K < 0 no agreement; K between 0-0.4: poor agreement; K between 0.4-0.6: fair agreement; K between 0.6-0.8: good agreement; K between 0.8-1: excellent agreement. Then, we calculated the rates of disease localisation in patients with and without a specific symptom. Comparisons among proportions were performed by Chi-squared test.

Univariate logistic regression analysis is used to examine the association of independent variable with one dichotomous dependent variable.

In our study we used this to evaluate the association of a specific symptom or multiple symptoms with a specific localisation of endometriotic lesions (single or in association). Data were expressed as an odds ratio and 95% interval of confidence (95% CI).

Finally, radar charts were designed to graphically express associations between symptoms and single compartments. A radar chart is a graphing technique used to simultaneously represent multivariate data in a single graph, using a series of spokes or rays projecting from a central point. Each ray represents a different variable, and the values of the variables are encoded into the lengths of the rays. We have six graphs, one for each symptom or associated symptoms studied. Each graph has six axes – each for every single compartment involved –ranging from OR of 0 to OR of 4, to display the most significant correlations between symptoms and compartments (Valensise et al., 2021). The further the distance of the colourful shape from the centre of the radar, the more important the symptom is in predicting endometriosis localisation.

## Results

To evaluate the interobserver agreement in describing pelvic endometriosis using #Enzian classification, 52 patients with ultrasound signs of endometriosis were evaluated by two different sonographers. The interobserver agreement is shown in Table I. K index ranged from 0.8 to1 for the most compartments with an excellent agreement between the two operators, except for compartment A and FA, which showed a good agreement (K index between 0.6-0.8).

**Table I:** Comparison between two different operators in the ultrasound evaluation of different #Enzian compartments. Cohen’s K value is used to evaluate the agreement rate between classifications: K < 0 no agreement; K between 0-0.4: poor agreement; K between 0.4-0.6: fair agreement; K between 0.6-0.8: good agreement; K between 0.8-1: excellent agreement. CI =confidence interval. USL: utero- sacral ligament.

Total n=52	**Observer A** Prevalence n (%)	**Observer B** Prevalence n (%)	**Match number n(%)**	**Cohen's kappa**	**95% CI**
A (vagina, rectovaginal space)	Total n=20 (38%)	Total n=27 (52%)	45(86%)	0.728	0.541 to 0.915
A1	6 (12%)	10 (19%)	48 (92%)	0.705	0.433 to 0.976
A2	12 (23%)	16 (31%)	48 (92%)	0.805	0.621 to 0.988
A3	2 (4%)	1 (2%)	50 (98%)	0.658	0.034 to 1.000
B right (USL, cardinal ligaments, pelvic sidewall)	Total n=10 (19%)	Total n=11 (21%)	51 (98%)	0.940	0.825 to 1.000
B1	1 (2%)	1 (2%)	52(100%)	1	1 to 1
B2	4 (8 %)	5 (9.6%)	51 (98%)	0.879	0.644 to 1.000
B3	5 (9.6%)	5 (9.6%)	52(100%)	1	1 to 1
B left (USL, cardinal ligaments, pelvic sidewall)	Total n=30 (57%)	Total n=33 (63%)	49 (92%)	0.880	0.748 to 1.000
B1	11 (21%)	12 (23%)	51 (98%)	0.944	0.836 to 1.000
B2	16 (30.7%)	17 (32.6%)	51 (98%)	0.956	0.870 to 1.000
B3	3 (5.7%)	4 (8%)	51 (98%)	0.847	0.554 to 1.000
C (rectum)	Total n=38 (73%)	Total n=36 (69%)	50 (96%)	0.906	0.779 to 1.000
C1	6 (11.5%)	4 (7.6%)	50 (96%)	0.779	0.483 to 1.000
C2	14 (27%)	16 (31%)	50 (96%)	0.906	0.779 to 1.000
C3	18 (34%)	16 (31%)	50 (96%)	0.913	0.794 to 1.000
O right (ovary)	Total n=11 (21%)	Total n=10 (19%)	51 (98%)	0.940	0.825 to 1.000
O1	3 (5.7%)	2 (3.8%)	51(98%)	0.790	0.392 to 1.000
O2	7 (13.4%)	7 (13.4%)	52 (100%)	1	1 to 1
O3	1 (2%)	1 (2%)	52 (100%)	1	1 to 1
O left (ovary)	Total n=11 (21%)	Total n=10 (19%)	51 (98%)	0.940	0.825 to 1.000
O1	5 (9.6%)	4 (8%)	51 (98%)	0.879	0.644 to 1.000
O2	6 (11.5%)	6 (11.5%)	52 (100%)	1	1 to 1
O3	0	0	/	/	/
T right (tubo-ovarian condition)	Total n=17 (32%)	Total n=16 (30%)	51 (98%)	0.956	0.870 to 1.000
T1	8 (15%)	7 (13.4%)	51 (98%)	0.922	0.772 to 1.000
T2	2 (3.8%)	2 (3.8%)	52(100%)	1	1 to 1
T3	7 (13.4%)	7 (13.4%)	52(100%)	1	1 to 1
T left (tubo-ovarian condition)	Total n=33 (63%)	Total n=34 (69%)	51 (100%)	0.958	0.877 to 1.000
T1	12 (23%)	12 (23%)	52(100%)	1	1 to 1
T2	1 (2%)	1 (2%)	52(100%)	1	1 to 1
T3	20 (38%)	21 (40%)	51 (98%)	0.960	0.882 to 1.000
FA (adenomyosis)	Total n=46 (88%)	Total n=43 (82%)	49 (94%)	0.766	0.514 to 1.000
FB (bladder)	Total n=6 (12%)	Total n=5 (10%)	51 (98%)	0.898	0.702 to 1.000
FI (intestine)	6 (12%)	6 (12%)	52(100%)	1	1 to 1
P (peritoneum)	0	0	/	/	/
FU (ureter)	0	0	/	/	/
FO (others)	0	0	/	/	/

Other 200 patients, all with ultrasound symptoms of endometriosis, were recruited to the study, and all received a TVS examination to classify pelvic endometriosis according to the #Enzian classification.

In our study population, the mean age of patients was 34.8 ± 7.3 (range 16 - 53); most of them were nulliparous (141/200, 70.5%); the main reasons that led women to undergo ultrasound examination were pelvic pain (dysmenorrhea, dyspareunia), infertility, bowel disorders, heavy menstrual bleeding, and ultrasound annual check. The most reported symptom was dysmenorrhea in 95% (190/200) of patients, followed by dyspareunia (45.5%, 91/200); approximately 45% of cases experienced HMB (90/200) and about 35% of patients complained of bowel symptoms (71/200).

Supplemental Table SI showed that the most involved compartments were B left (64%); FA (55.5%), T left (55.5%), and O left (49%). In our population, compartment B left was more represented compared to B right (p<0.0001), as for compartment T left compared to T right (p=0.01); no statistically significant differences were noticed comparing compartments O right and left.

**Table SI:** Ultrasound distribution of #Enzian compartments among the 200 patients of the study population. USL: uterosacral ligament.

**#Enzian Compartments**	**Total patients (200)** Prevalence n(%)
A (vagina, rectovaginal space)	Total n=69 (34.5%)
A1	39 (56.5%)
A2	27 (39.1%)
A3	3 (4.3%)
B right (USL, cardinal ligaments, pelvic sidewall)	Total n=62 (31%)
B1	39 (62.9%)
B2	21 (33.9%)
B3	2 (3.2%)
B left (USL, cardinal ligaments, pelvic sidewall)	Total n=128 (64%)
B1	90 (70.3%)
B2	38 (29.7%)
B3	0 (0.0%)
C (rectum)	Total n=65 (32.5%)
C1	10 (15.4%)
C2	30 (46.1%)
C3	25 (38.5%)
O right (ovary)	Total n=81 (40.5%)
O1	53 (65.4%)
O2	25 (30.9%)
O3	3 (3.7%)
O left (ovary)	Total n=98 (49%)
O1	57 (58.2%)
O2	33 (33.7%)
O3	10 (10.2%)
T right (tubo-ovarian condition)	Total n=86 (43%)
T1	20 (23.2%)
T2	36 (41.9%)
T3	29 (33.7%)
T left (tubo-ovarian condition)	Total n=111 (55.5%)
T1	26 (23.4%)
T2	50 (45%)
T3	37 (33.3%)
FA (adenomyosis)	Total n=111 (55.5%)
FB (bladder)	Total n=3 (1.5%)
FI (intestine)	Total n=9 (4.5%)
P (peritoneum)	0 (0.0%)
FU (ureter)	0 (0.0%)
FO (others)	0 (0.0%)

The small sample of data, and therefore distribution between the grades of endometriotic lesions, did not allow us to perform a statistical analysis separately for each grade.

The compartments that had left and right laterality were considered as one compartment in the further statistical analysis.

In our study population only five patients complained of dysuria, of which only one showed deep endometriosis of the bladder; therefore, due to the low number of cases, we did not include this symptom in the statistical analysis.

Since dysmenorrhea was present in almost all selected populations (190/200, 95%), it did not show any statistical association with specific endometriosis localisation. Therefore, all the symptoms that we analysed in the further statistical analysis should be considered associated with dysmenorrhea.

Table II shows the comparison between patients with and without specific symptoms and percentages of compartments involved; a p-value <0.05 was considered statistically significant.

**Table II:** A Chi-squared test was performed to compare endometriosis patients (all with dysmenorrhea) with and without other specific symptoms (yes vs no) and relative #Enzian compartments involved. Data are shown as n (number of patients) and percentages (%). *p<0.05. Bowel sympt: bowel symptoms. Dysp: dyspareunia. HMB: heavy menstrual bleeding.

		#Enzian Compartments
Symptoms		A (69)	B (160)	C (65)	O (45)	T (132)	FA (110)	O+T (26)	O+C (17)	A+C (33)	B+C (9)	A+T (35)	A+O (33)	B+O (10)	B+A (16)	A+FA (42)	B+FA (22)	C+FA (46)	T+FA (47)	O+FA (25)	A+B+FA (12)
Type	Present	n (%)	n (%)	n (%)	n (%)	n (%)	n (%)	n (%)	n (%)	n (%)	n (%)	n (%)	n (%)	n (%)	n (%)	n (%)	n (%)	n (%)	n (%)	n (%)	n (%)
DYSP	Yes (91)	34(37)	22(24)*	28(31)	12(13)	34(37)	57(63)	8(9)	4(4)	16(18)	7(8)	19(21)	5(5)	6(7)	12(13)*	23(25)	17(19)*	21(23)	29(32)*	7(8)	10(11)*
	No (109)	35(32)	12(11)	37(34)	33(30)	32(29)	54(50)	18(17)	13(12)	17(16)	3(3)	16(15)	13(12)	5(5)	4(4)	9(17)	6(6)	25(23)	19(17)	18(17)	2(2)
BOWEL SYMPT	Yes (71)	29(41)	18(25)*	28(39)	17(24)	27(38)	41(58)	10(14)	8(11)	17(24)*	6(8)	15(21)	6(8)	6(8)	8(11)	19(27)	12(17)	20(28)	22(31)	12(17)	7(10)
	No (129)	40(31)	16(12)	37(29)	28(22)	39(30)	70(54)	23(14)	9(7)	16(12)	4(3)	20(16)	12(9)	5(4)	8(6)	23(18)	11(9)	26(20)	26(20)	13(10)	5(4)
HMB	Yes (90)	26(29)	15(17)*	29(32)	20(22)	31(34)	58(64)*	10(11)	6(7)	14(16)	8(9)*	15(17)	6(7)	5(6)	8(9)	18(20)	12(13)	24(27)	24(27)	12(13)	7(8)
	No (110)	43(39)	19(17)	36(33)	25(23)	35(32)	53(48)	16(15)	11(10)	19(17)	2(2)	20(18)	12(11)	6(5)	8(7)	24(22)	11(10)	22(20)	24(22)	13(12)	5(5)
DYSP+HMB	Yes (49)	18(37)	12(24)	16(33)	8(16)	18(37)	32(65)	5(10)	3(6)	9(18)	6(12)*	11(22)	4(8)	4(8)	7(14)	13(27)	10(20)*	13(27)	15(31)	5(10)	6(12)*
	No (151)	51(34)	22(15)	49(32)	37(25)	48(32)	79(52)	21(14)	14(9)	24(16)	4(3)	24(16)	14(9)	7(5)	9(6)	29(19)	13(9)	33(22)	33(22)	20(13)	6(4)
DYSP+BOWEL SYMPT	Yes (37)	17(46)	14(38)*	13(35)	8(22)	17(46)	22(59)	6(16)	3(8)	10(27)	5(14)*	11(30)*	4(11)	6(16)	7(19)*	12(32)	10(27)	10(27)	15(41)*	6(16)	6(16)*
	No (163)	52(32)	20(12)	52(32)	37(23)	49(30)	89(55)	20(12)	14(9)	23(14)	5(3)	24(15)	14(9)	5(3)	9(6)	30(18)	13(8)	36(22)	33(20)	19(12)	6(4)
HMB+BOWEL SYMPT	Yes (37)	12(32)	10(27)	14(38)	10(27)	16(43)	27(73)*	5(14)	4(11)	7(19)	5(14)*	9(24)	3(8)	4(11)	6(16)*	10(27)	7(19)	13(35)	15(41)*	8(22)	5(14)*
	No (163)	57(35)	24(15)	51(31)	35(21)	50(31)	84(52)	21(13)	13(8)	26(16)	5(3)	26(16)	15(9)	7(4)	10(6)	32(20)	16(10)	33(20)	33(20)	17(10)	7(4)

Analysing statistically these associations we noticed that dyspareunia is more often reported by patients with lesions in compartment B (22/91, 24%); B compartment is also associated with the presence of bowel symptoms (18/71, 25%); whereas patients with HMB showed a higher percentage of adenomyosis (58/90, 64%).

We then proceeded to analyse statistically the association between single symptoms and combined compartments; a higher percentage of combined affected compartments was detected, B+A (12/91, 13%), B+FA (17/91, 19%), and T+FA (29/91, 32%) in patients with dysmenorrhea and dyspareunia. Furthermore, patients with bowel symptoms and dysmenorrhea showed lesions more frequently in compartments A+C (17/71, 24%) and HMB is more often correlated with compartments B+C both affected (8/90, 9%).

In further evaluation of combined symptoms associated with a single compartment (Table II) we observed that the B compartment showed a significant percentage when bowel symptoms are associated with dyspareunia (14/37, 38%). Patients with HMB and bowel symptoms are at higher risk of presenting FA (27/37, 73%).

To confirm the association between symptoms and localisations, we proceeded to perform the univariate logistic regression analysis; statistically significant correlations are shown in Table III. Data were expressed as Odds Ratio and confidence interval (95% IC).

**Table III:** Univariate logistic regression analysis of specific symptoms and #Enzian compartments. All symptoms must be considered together with dysmenorrhea. Data are shown as Odds Ratio and 95% interval of confidence (95% CI OR). *p<0.05. Bowel Sympt: bowel symptoms. Dysp: dyspareunia. HMB: heavy menstrual bleeding

SYMPTOMS	#Enzian Compartments																			
	A	B	C	O	T	FA	O+T	O+C	A+C	B+C	A+T	A+O	B+O	B+A	A+FA	B+FA	C+FA	T+FA	O+FA	A+B+FA
Dysp	1.26 (0.70-2.26)	2.58* (1.19-5.56)	0.86 (0.48-1.57)	0.35 (0.17-0.63)	1.43 (0.79-2.59)	1.71 (0.97-3.01)	0.49 (0.20-0.18)	0.34 (0.17-1.08)	1.15 (0.55-2.44)	2.94 (0.74-11.7)	1.53 (0.74-3.19)	0.43 (0.15-1.25)	1.47 (0.43-4.98)	3.99* (1.24-12.8)	1.60 (0.81-3.18)	3.94* (1.48-10.4)	1.01 (0.52-1.95)	2.22* (1.14-4.30)	0.42 (0.17-1.06)	6.60* (1.41-30.9)
Bowel sympt	1.54 (0.84-2.81)	2.40* (1.14-5.07)	1.62 (0.88-2.98)	1.14 (0.57-2.26)	1.42 (0.77-2.70)	1.15 (0.64-2.07)	1.16 (0.49-2.71)	1.69 (0.62-4.60)	2.22* (1.04-4.73)	2.88 (0.79-10.5)	1.46 (0.69-3.07)	0.90 (0.32-2.51)	2.29 (0.67-7.79)	1.92 (0.69-5.36)	1.68 (0.84-3.36)	2.18 (0.91-5.24)	1.55 (0.79-3.04)	1.78 (0.92-3.45)	1.81 (0.78-4.22)	2.71 (0.83-8.89)
HMB	0.63 (0.35-1.15)	0.96 (0.46-2.01)	0.98 (0.54-1.77)	0.97 (0.50-1.89)	1.13 (0.62-2.03)	1.95* (1.10-3.45)	0.73 (0.32-1.71)	0.64 (0.23-1.81)	0.88 (0.41-1.88)	5.27* (1.09-25.04)	0.90 (0.43-1.88)	0.58 (0.21-1.62)	1.02 (0.30-3.46)	1.24 (0.45-3.46)	0.90 (0.45-1.78)	1.38 (0.58-3.31)	1.45 (0.75-2.82)	1.30 (0.68-2.50)	1.15 (0.50-2.66)	1.77 (0.58-5.78)
Dysp + HMB	1.14 (0.58-2.23)	1.90 (0.86-4.20)	1.01 (0.51-2.01)	0.60 (0.26-1.40)	1.25 (0.63-2.44)	1.71 (0.88-3.35)	0.70 (0.25-1.98)	0.64 (0.17-2.32)	1.19 (0.51-2.77)	5.13* (1.38-19.0)	1.53 (0.69-3.41)	0.87 (0.27-2.78)	1.83 (0.51-6.53)	2.63 (0.92-7.48)	1.52 (0.72-3.22)	2.72* (1.11-6.68)	1.29 (0.61-2.71)	1.58 (0.77-3.24)	0.74 (0.26-2.10)	3.37* (1.03-10.1)
Dysp + Bowel sympt	1.81 (0.88-3.75)	4.35* (1.93-9.81)	1.16 (0.55-2.45)	0.94 (0.40-2.23)	1.98 (0.5-4.10)	1.22 (0.59-2.52)	1.38 (0.51-3.73)	0.94 (0.25-3.45)	2.25 (0.96-5.27)	4.94* (1.35-18.0)	2.45* (1.07-5.70)	1.29 (0.40-4.17)	6.12* (1.76-21.3)	3.99* (1.38-11.5)	2.13 (0.96-4.71)	4.27* (1.70-10.7)	1.31 (0.58-2.95)	2.69* (1.26-5.74)	1.47 (0.54-3.97)	5.06* (1.53-16.7)

We noticed that when we evaluated patients who complained of more symptoms, we detected more statistical significance with the association of compartments involved.

Dyspareunia and HMB occurred more frequently in patients with dysmenorrhea and lesions on compartments B+C (p=0.01), and B+FA (p=0.03).

Patients with dysmenorrhea, dyspareunia, and bowel symptoms showed the greatest variety of localisation of the disease. We observed associations with compartments B+O (p<0.01), B+C (p=0.02), B+A (p=0.01), B+FA (p<0.01), A+T (p=0.04) and T+FA (p=0.01).

Regarding the combination HMB and bowel symptoms, we observed a statistically significant association with the following associated compartments: B+C (p=0.02), B+A (p=0.05), and T+FA (p=0.01).

Finally, the correlation between associated symptoms with more than two compartments involved was assessed. Compartments A, B, and FA were combined based on the previous statistical significances, and patients with dyspareunia were observed to have a higher risk of presenting lesions of these compartments together (p<0.01). The association of A+B+FA is also significantly present in patients with dyspareunia and HMB (p=0.05), dyspareunia and bowel symptoms (p=0.01), and HMB and bowel symptoms (p=0.05).

An attempt was made to correlate the presence of a symptom with the dimension of the lesion (grades 1-3); there is no statistically significant difference observed between the presence of symptoms and the size of an endometrioma, neither with the size of a rectal nodule or compartment B nor with respect to adhesions.

**Figure 1:**
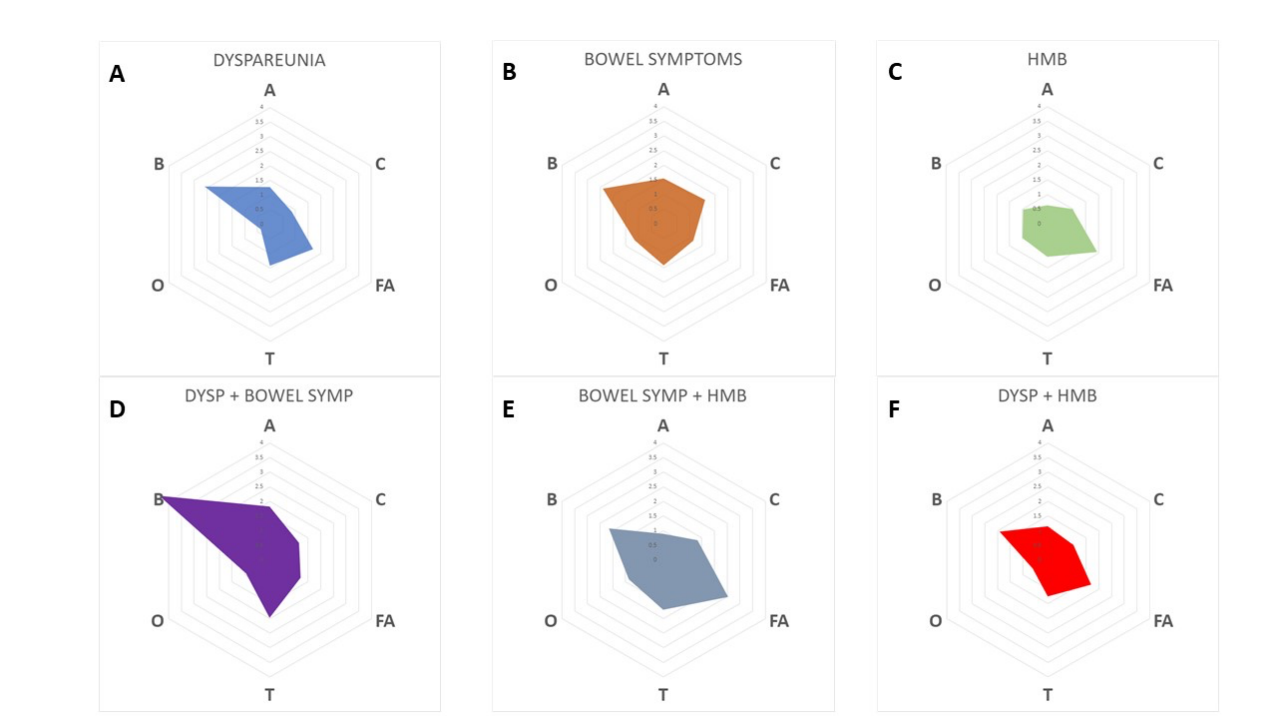
Radar Charts for the association between single and associated symptoms and single #Enzian compartments involved. All symptoms must be considered together with dysmenorrhea. Note the association between compartment B and dyspareunia (1A) and dyspareunia plus bowel symptoms (1D). In figure 1C, note the correlation between HMB and adenomyosis (FA). Bowel Sympt: bowel symptoms. Dysp: dyspareunia.

Finally, to summarise our results we focused on Figure 1 and Table IV to analyse the most significant associations between symptoms and compartments involved to guide the sonographer.

**Table IV:** Most significant associations between symptoms and #Enzian compartments (single and combined) involved. All symptoms must be considered together with dysmenorrhea. Bowel Sympt: bowel symptoms. Dysp: dyspareunia. HMB: heavy menstrual bleeding.

	#Enzian Compartments involved	
Symptoms	Single	Combined
DYSP	B	B+A, B+FA, T+FA, A+B+FA
BOWEL SYMPT	B	A+C
HMB	FA	B+C
DYSP + HMB		B+A, B+FA, A+B+FA
DYSP+ BOWEL SYMPT	B	B+C, B+O, A+T, B+A, B+FA, T+FA, A+B+FA
HMB + BOWEL SYMPT	FA	B+A, B+C, T+FA, A+B+FA

In Figure 1, radar charts were designed to express the association between symptoms, single and combined compartments, and single compartments. The association between dyspareunia and compartment B (Figure 1A) and HMB and adenomyosis (Figure1C) can be observed; there is also strong association of compartment B when dyspareunia is associated with bowel symptoms (Figure 1D).

In Table IV we have represented all the significant statistical associations, also including multiple compartments.

## Discussion

This study demonstrated that the reproducibility of #Enzian classification was high among different operators using TVS and some symptoms correlated to specific #Enzian compartments.

The main objective of this study was to correlate the patients’ reported symptoms with specific localisation of pelvic endometriosis by non-invasive ultrasound examination, avoiding the bias due to previous surgical adhesions, incomplete surgery, or symptomatic relief due to hormonal treatment. We utilised the #Enzian score to describe the disease extension since it divides the pelvis into different compartments and grades the disease according to the sizes of the lesions. Recent studies confirmed the accuracy of TVS examination in detecting pelvic endometriosis using #Enzian classification (Di Giovanni et al., 2022; Montanari et al., 2022; Bindra et al., 2023).

To confirm the reproducibility of TVS non- invasive diagnosis throughout #Enzian classification, we performed an interobserver study which showed high agreement in the compartments assessment. Indeed, K Cohen’s results in Table I showed a very low discrepancy (excellent agreement) rate between the two different operators in detecting endometriotic lesions. Only compartments A and FA showed a lower agreement (good agreement), which may be because vaginal endometriotic foci and mild adenomyosis can be difficult to detect by TVS.

In our study population, dysmenorrhea was noted by 95% of patients: for this reason, it did not correlate with any specific #Enzian compartment, thus arising as a non-specific symptom of particular endometriotic lesions. Therefore, our statistical analysis was performed considering the other symptoms in patients having dysmenorrhea.

Dyspareunia has been shown to be the most accurate symptom in describing the presence of specific endometriotic lesions, along with dysmenorrhea; specifically, patients with dyspareunia and dysmenorrhea showed a two times greater probability of having a lesion of the uterosacral ligaments (compartment B); this probability doubled if the lesion on the uterosacral ligaments was associated with a lesion of compartment A, becoming even higher in case of concomitant adenomyosis. These results could explain why retrocervical endometriosis, involving the torus, vagina, recto-vaginal septum, and uterosacral ligaments is the most painful localisation of the disease regardless of the lesion dimensions. In accordance with previous literature, in our population HMB was significantly associated with adenomyosis (Exacoustos et al., 2020).

Bowel symptoms were associated with lesions on utero-sacral ligaments. Moreover, by combining bowel symptoms with dysmenorrhea and dyspareunia we obtained the highest number of compartments involved; therefore, these symptoms should be carefully investigated during a gynaecological pre-ultrasound evaluation.

We also decided to evaluate the association between symptoms, since most of the patients complained of multiple symptoms. Moreover, the disease often does not occur as a single lesion but as multiple lesions. The analysis showed how the presence of dysmenorrhea, dyspareunia, and bowel symptoms, was associated to lesions in the retrocervical area, whereas the presence of HMB should lead to an accurate myometrial evaluation with 2D and 3D ultrasound in order to detect any ultrasound signs of adenomyosis.

We noticed also that bowel symptoms and HMB were associated with the presence of lesions on compartment B and adenomyosis (Figure 1E). This means that the uterus and retrocervical area could explain the presence of bowel symptoms; additionally, adenomyosis could be caused by the infiltration of posterior DIE.

The association of TVS findings described by the #Enzian classification and symptoms could lead to a new concept of disease severity. Small lesions of the retrocervical area with severe and combined symptoms, which have an impact on the patient’s quality of life can be considered severe disease, whereas asymptomatic bowel lesions, also more than 3 cm in length (C3), could be considered a mild disease. This concept is crucial in an era in which the diagnosis of endometriosis is no longer just surgical. With the help of TVS as a non-invasive diagnostic tool, we can change the classification and management of the disease.

The importance of having selected patients who have never undergone abdominal and pelvic surgery and who have not undergone medical hormonal therapy for at least six months, has allowed us to study a clean cohort, without interference with the symptoms.

This study has some limitations. The first limit could be considered the sample size: a larger number of cases would be needed to confirm our data on the association between symptoms and compartments.

In our cohort, ovarian endometriosis did not show any statistical association with specific symptoms or combined symptoms. This result could be explained by the high percentage (nearly 50%) of ovarian involvement (mostly left endometrioma). Therefore, as well as dysmenorrhea, was found to be a non-specific symptom of endometriosis, also having an ovarian localisation could be a nonspecific localisation to develop a symptom. The same could be stated for compartment T since adhesions are very frequent in patients with endometriosis.

Finally, in our study population, the correlation between the symptoms and the grades of endometriotic lesions was not possible considering the low number of patients who presented certain grades of lesion. Therefore, these data will need to be further investigated by increasing the sample size, or in a subsequent multi-centre study.

In the literature only a few papers described the association between the severity of symptoms and the spread of endometriotic lesions; Montanari et al. (2019) described statistically significant correlations between symptom severity of dyschezia and lesion size in compartment C and severity of dyspareunia and lesion size in compartment B. Severity of dysmenorrhoea was also correlated with lesion size in compartment A and was associated with the presence of adenomyosis. (Montanari et al., 2020)

Kor et al. (2020) found that the cumulative size of posterior deep infiltrative endometriosis (DIE) (less than 1 cm) is significantly correlated with minimal severity of dyspareunia and chronic pelvic pain. The incidence of dyspareunia was more prevalent in patients with complete stenosis of the Douglas pouch than those with incomplete stenosis (Kor et al., 2020)

## Conclusions

In conclusion, our study showed that the #Enzian classification is easy to use and reproducible between different operators by using TVS. Patients’ symptoms should be accurately investigated to guide ultrasound detection of pelvic disease localisations. Dysmenorrhea is a non-specific symptom of endometriosis localisations, while other endometriosis-related symptoms seem to be more specific in helping the sonographer to evaluate #Enzian compartments. The association between symptoms and #Enzian compartments could give the clinician a new concept of the severity of the disease to better classify patients with endometriosis, establishing the best therapeutic management.
